# Change in Sleep Quality of Residents the Night Before High-Fidelity Simulation: Results From a Prospective 1-Year National Survey

**DOI:** 10.5152/TJAR.2022.21235

**Published:** 2022-08-01

**Authors:** Marion Calmettes, Lucas Denoyel, Antoine Duclos, Corinne Lejus-Bourdeau, Antonia Blanié, Caroline Forestier, Floriane Ciceron, Laurent Mattatia, Clément Buleon, Cédric Damm, Gilles Mahoudeau, Jean-Jacques Lehot, Thomas Rimmelé, Marc Lilot

**Affiliations:** 1 Department of Anaesthesiology and Reanimation, Hospices Civils de Lyon, Lyon, France; 2 Lyonnais Center for Education by Simulation in Health, Claude Bernard Lyon 1 University, SAMSEI, Lyon, France; 3 Department of Health Data, Hospices Civils de Lyon, Lyon, France; 4 Research on Healthcare Performance (RESHAPE), Claude Bernard Lyon 1 University, Lyon, France; 5 Department of Anaesthesia and Surgical Resuscitation, Hôtel Dieu, Nantes University Hospital, Nantes, France; 6 Department of Anaesthesiology and Surgical Resuscitation, CHU Bicêtre LabForSIMS Faculty of Medicine, Paris-Sud, Le Kremlin Bicêtre, France; 7 CEnSIM Savoie Emergency Care - Savoie Simulation Education Center, Savoie Metropole Hospital Center, France; 8 Medsim Simulation Center, University of Franche Comté, France; 9 Department of Anaesthesia, Reanimation and Pain Emergency, SIMHU-Nîmes Medical Simulation Center, Carémeau Hospital Group, Nîmes University Hospital, France; 10 Normandy Health Simulation Center (NorSimS), Caen University Hospital, France; 11 Emergency Care Teaching Center, CESU, University Hospital Center of Rouen, France; 12 UNISIMES European Health Simulation Unit, Strasbourg University Faculty of Medicine, France; 13 University of Claude Bernard Lyon 1-Bio mericux-Hospices Civils de Lyon, Lyon, France

**Keywords:** Education, high-fidelity simulation, Leeds Sleep Evaluation Questionnaire, sleep disturbance

## Abstract

**Objective:**

The stress level of participants in high-fidelity simulation stems from various factors but may result in anticipatory anxiety causing sleep disturbances during the night prior to simulation. The objective of this survey was to determine the change in sleep quality of residents during the night prior to the simulation.

**Methods:**

The survey was proposed for 1 year to all residents at the beginning of the simulation, in 10 simulation centres. The questionnaire combined demographics and the Leeds Sleep Evaluation Questionnaire using visual analogue scales divided into 4 sleep qualitative domains. The primary outcome was the prevalence of sleep disturbance (>10 mm on 1 domain). Secondary outcomes were the prevalence of severe sleep disturbance (>25 mm), as well as qualitatively and quantitatively reported explanatory sleep parameters.

**Results:**

Among respondents, 66% [95% CI: 63 to 69] of residents had more than 10 mm and 27% [95% CI: 24 to 30] had more than 25 mm of sleep disturbance. Residents with a sleep disturbance of more than 10 mm had fewer hours of sleep (6.4 [standard deviation = 1.8] vs 7.3 [standard deviation = 1.3], difference: −0.9 [95% CI: −1.1 to −0.7]; *P* < .0001), with a higher number of night-time awakenings (1.3 [standard deviation = 1.5] vs 0.7 [standard deviation = 0.9], difference: 0.6 [95% CI: 0.4 to 0.8]; *P* < .0001).

**Conclusion:**

Among residents participating in the simulation, a high prevalence of change in sleep quality during the night before the simulation was noted. Strategies to help residents achieve better sleep prior to simulation should be explored.

**Main Points**

- Two-thirds of residents had sleep disturbance prior to high-fidelity simulation (HFS).
- Residents with a sleep disturbance had fewer hours of sleep.
- Strategies to help residents achieve better sleep prior to HFS should be explored.

## Introduction

High-fidelity simulation (HFS) offers opportunities to practice high-risk and rare situations in a safe training environment. Moreover, HFS has proven its efficacy to enhance learning acquisition, justifying the significant integration of HFS into resident medical education curriculums.^1^ High-fidelity simulation strives to reproduce ultrarealistic-simulated healthcare theatres to help active participants perform in simulation as if they were facing real situations. The active learning environment in HFS has been reported to generate stress through several identified causes such as the fear of making mistakes or the fear of negative evaluation by instructors or peers.^2,3^ Anticipated fears might disturb the quality of sleep during the night before HFS.^4^ Furthermore, residents are at risk for chronic and acute sleep disorders. Shift working can lead to sleep deprivation, which can affect cognitive performance, vigilance, memory, and non-technical skills.^5-8^ This might result in impaired performance during practice and more medical errors.^9^ The aim of this survey was to assess the day of the HFS and the prevalence of change in sleep quality during the night prior to HFS.

## Methods

The study protocol was approved a priori by the scientific committee of the Société Francophone de Simulation en Santé, by the Ethics Committee of Hospices Civils de Lyon (30092016) and was pre-registered in clinicaltrial.gov (NCT02922608).

This prospective survey was administered to all residents directly at the beginning of the HFS session from November 2016 to November 2017 in 10 participating university medical simulation centres. Another questionnaire was sent to each participating simulation centre to collect information on their local organization of HFS.

The HFS sessions in the different centres were similar: HFS were scheduled on weekdays from Monday to Friday and on daily hours and the duration of the session was 7 hours on average, including 5-14 participants for each session. The number of HFS sessions was 1-5 per year per resident. The HFS was mandatory for residents but without evaluation for to be graduate or evaluation for certification. All learners had preliminary information available on a website at their disposal. In all centres, scenarios were simultaneously video transmitted to observe residents. Scenarios lasted approximately 15 minutes followed by 30 minutes of debriefing. Three instructors were involved in each HFS in all centres.

All residents who participated in HFS were asked to complete the survey upon their arrival at the simulation centre. Therefore, the survey was completed by each resident on the day of the HFS. No exclusion criteria were applied. Sleep deprivation was evaluated with the Leeds Sleep Evaluation Questionnaire (LSEQ) which was developed to monitor sleep disturbances during psychopharmacological investigations.^10^ The LSEQ is currently used in non-pharmacological settings and in clinical research to measure sleep quality.^10-12^

**Supplementary Figure 1.**
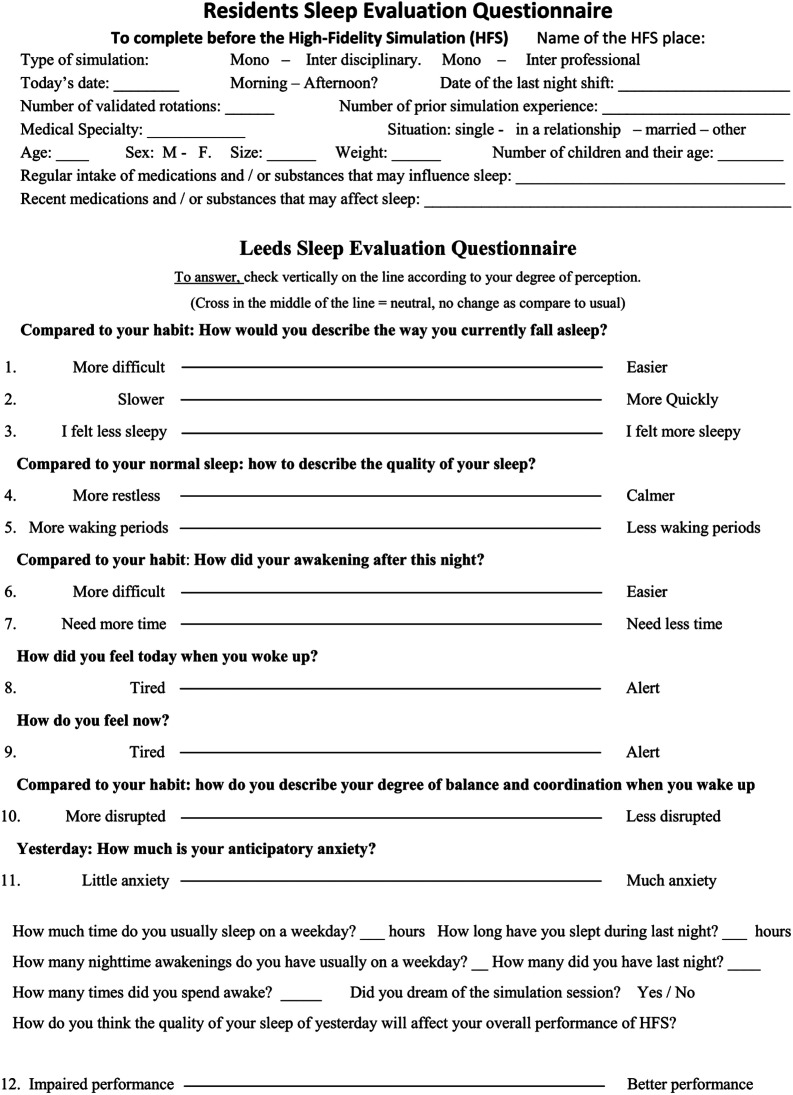
Survey questionnaire with the Leeds Sleep Evaluation Questionnaire

The questionnaire was anonymous and included numerous demographic data. The LSEQ and additional quantitative questions related to normal sleep and sleep during the previous night were collected (Supplementary Figure 1). The LSEQ is a standardized self-reporting questionnaire comprising 10 consecutive visual analogue scales (VAS) of 100 mm. The middle of the scale (50 mm) corresponds to the absence of disturbance as compared to usual sleep. The 10 VAS were divided into 4 different qualitative sleep domains: ease of getting to sleep (questions 1-2-3), perceived quality of sleep (questions 4-5), ease of awakening from sleep (questions 6-7), and integrity of behaviour following wakefulness (questions 8-10).^9^ The mean of VAS for each qualitative domain was calculated to assess the sleep disturbance. A difference of 10 mm (i.e., score < 40 mm: 20% of sleep disturbance) for one or more domains of LSEQ was considered clinically relevant to state significant changes in sleep quality.^13^ This disturbance of 10 mm was considered a sleep disturbance. A difference greater than 25 mm (i.e., score < 25 mm: 50% of sleep disturbance) for one or more domains of LSEQ was considered a severe sleep disturbance.

Additional questions explored information about normal sleep and sleep during the previous night: number of hours of sleep, number of night-time awakenings, and the time spent awake. Residents were asked if they dreamed about the HFS the night before. The overall HFS anticipatory anxiety and the impact of sleep disturbance on the upcoming HFS performance were shown with specific VAS. Additional factors were surveyed including parental status, medications, time from the last night shift, number of prior HFS, and number of validated rotations. In France, the residency is made of ten 6-month rotations. High-fidelity simulation was then performed at the discretion of each simulation centre following the national guidelines for simulation in healthcare.^14^

In order to compare the experimental data with the normal rate of sleep disturbance among residents, the same questionnaire was distributed to a sample of 50 residents via the information provided by email and provided in the medical centre, a few months after the HFS. This sample of residents includes residents in anaesthesia and intensive care from the main investigator medical centre. They were asked to complete the questionnaire when they had not had a night shift for at least 3 days prior. The response was anonymous and returned by email or via the secretary of anaesthesia and intensive care departments.

The primary endpoint was the prevalence of sleep disturbance. The secondary endpoints were the incidence of severe sleep disturbance, sleep duration time, number of night-time awakenings, time spent awake, and prevalence of dreams about HFS.

### Statistical A nalysis

Distributions were assessed by Kolmogorov–Smirnov test. Categorical variables were presented using absolute and relative frequencies and compared using the *χ* ^2^ test or Fisher’s exact test as appropriate. Continuous variables were described using mean (standard deviation) or median (25th-75th percentile) and compared using Student’s *t*-test or Mann–Whitney as appropriate. Difference estimates with 95% CI are provided. The associations were quantified by odds ratios with their 95% CI. For the construction of multivariate models, factors with *P*-values less than .20 were included in a logistic regression analysis. Two logistic regression models were built, one with the quantitative variables expressed as continuous variables and one with the quantitative variables categorized as dichotomous variables according to their optimal threshold value. The optimal threshold value was determined by constructing receiver operating characteristic curves for predicting sleep disturbance.

All tests were 2-tailed, and *P* < .05 was considered statistically significant. Data analyses were performed using MedCalc® version 12.1.4.0 for Windows (Medcalc Software, Ostend, Belgium) and Statistica® version 6.0 (Statsoft, Tulsa, Okla, USA).

## Results

Ten medical simulation centres in France participated in the study. The survey included 1112 questionnaires from November 2016 to November 2017. No questionnaire was excluded. This cohort was represented mainly by residents in anaesthesiology and intensive care speciality (n = 661: 59.6%). Characteristics of residents are presented in Table 1.

### Primary Endpoint

**Figure 1.**
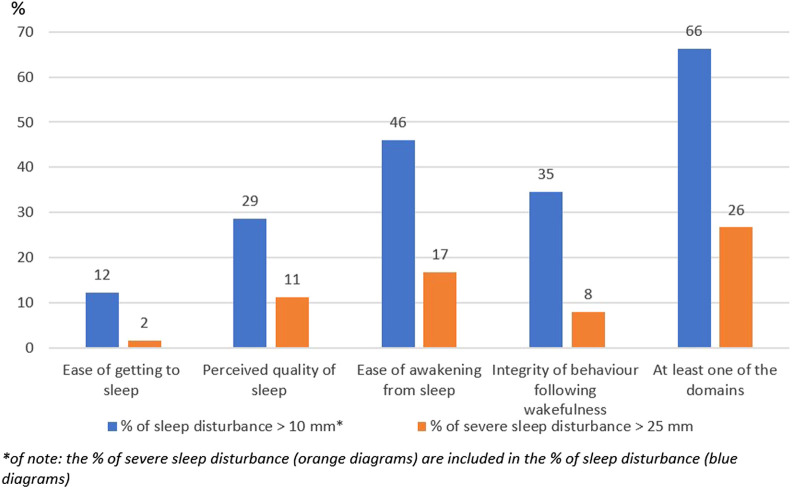
Prevalence of sleep disturbance for the 4 domains of Leeds Sleep Evaluation Questionnaire (LSEQ).

The prevalence of sleep disturbance and severe sleep disturbance was respectively 66% [95% CI: 63-69] and 27% [95% CI: 24-30] (Figure 1).

### Secondary Endpoints

The normal sleep duration was similar between residents without sleep disturbance and residents who experienced sleep disturbance (difference: −0.03 [95% CI: −0.14 to 0.08]; *P* = .628) or severe sleep disturbance (difference: −0.5 [95% CI: −0.17 to 0.06]; *P* = .358) (Table 2 and 3).

Residents experiencing sleep disturbance slept 1 hour less the night prior to HFS as compared to normal sleep (6.4 vs 7.3 hours [95% CI: −1.1 to −0.7]; *P* < .0001). They had more night-time awakenings (1.3 vs 0.7 [difference: 0.6 (95% CI: 0.41 to 0.75); *P* < .0001]) and the time spent awake was more important (12 (25) vs 6 (14) minutes (difference: 5.5 [95% CI: 2.8 to 8.3]; *P* < .0001) the night before HFS (Table 2).

Residents with severe sleep disturbance also slept 1 hour less the night prior to HFS (5.8 vs 7.0, difference: −1.2 [95% CI: −1.4 to −0.9]; *P* < .0001). They had more night-time awakenings with more time spent awake (Table 3).

There were fewer females with sleep disturbance than in the group without sleep disturbance (49% vs 57% *P* = .015). No association between HFS experience, age, number of validated rotations (from 0 to 10 semesters), and sleep disturbance the night prior to HFS was observed (Table 2).

Residents with sleep disturbance and severe sleep disturbance had more anticipatory anxiety about HFS (respectively 41 vs 34, difference: −7.3 [95% CI: −4.1 to −10.4]; *P* < .001), and 42 vs 38, difference −4.2 [95% CI: −0.9 to −7.6]; *P* = .13). They also perceived that their performance would be more impaired because of the sleep the night before than in groups without sleep disturbance: (42 mm vs 53 mm (difference: −11.5 mm [95% CI:−13 to −10]; *P* < .0001) and 36 mm vs 49 mm (difference: −13.5 mm [95% CI: −15.2 to −11.8]; *P* < .001), respectively). There were 264 (24%) residents with night on duty during the 72 hours before the HFS. In the univariate analysis, residents with sleep disturbance and severe sleep disturbance did not have difference in the delay since the last night on duty as compared with residents without sleep disturbance. The logistic regression model did not select this factor as pertinent for the multivariate analysis (Table 2 and 3).

Multivariate analysis found that being female (OR: 0.7, 95% CI: 0.49-0.99; *P* = .046) is an independent protective factor for sleep disturbance. Having higher anticipatory anxiety the night before the HFS (best threshold observed was >46 mm) was also found to be an independent factor of sleep disturbance (OR: 1.72, 95% CI: 1.29-2.29; *P* = .0002) (Table 4).

In line with the VAS-anticipatory anxiety, the anticipation of better HFS performance with better quality of sleep (best threshold observed was >46 mm) was found to be an independent protective factor for sleep disturbance (OR: 0.16; 95% CI: 1.29-2.29; *P* < .0001) (Table 4).

For the sample of residents who filled out the questionnaire well after the HFS, the prevalence of sleep disturbance and severe sleep disturbance was lower (respectively, 8%, 95% CI: 2-19; *P* < .001 and 8%, 95% CI: 2-19; *P* = .005).

## Discussion

This multicentre study found a high prevalence (>60%) of sleep disturbance among residents the night before an HFS session, as compared to the sleep habits shown by the LSEQ. The total sleep time was also impaired, with a 1-hour reduction as compared to baseline, an increase in the frequency of nocturnal awakenings, and a two-fold longer time spent awake during the night. The residents were also more anxious on the night before HFS and they thought that their performance would be impaired by the sleep disturbance on the night before. This high prevalence of sleep disturbance prior to HFS was not observed in the control group. Therefore, one might suspect that HFS could be the direct cause of the sleep disturbance the night before.

No association between HFS experience, age, number of validated rotations, and sleep disturbance the night before the HFS session was observed. Surprisingly, being on call in the previous days was not found to be a risk factor for sleep disturbance. Residents on call within the 3 days prior to HFS were not excluded from the analysis. Being female was found to be associated with less sleep disturbance in this survey, in both univariate and multivariate analysis. This finding conflicts with existing literature as women have been reported to be more affected by sleep disturbance than men. Indeed, a meta-analysis found that women were more predisposed to insomnia.^15^ A study that assessed sleep with home monitoring described that women go to bed and fell asleep earlier, that the sleep period time was markedly longer for women, and that women reported more awakenings, more total time spent awake during the night, and poorer sleep quality.^16^

The present survey showed that residents who had sleep disturbance and severe sleep disturbance had more anticipatory anxiety the night before HFS. The high realism of the simulation session can lead to anticipatory anxiety because of several parameters. The stress of being observed and evaluated, fear of failure or of being judged by peers, or performance pressure could explain sleep disturbance the night before HFS. Furthermore, medical residents are even more exposed to acute and chronic sleep disorders. Numerous studies have found a high rate of sleep disturbance among medical and university residents, from 1.5% to 40%, assessed by the Pittsburgh Sleep Quality Index (PSQI).^17,18^ Long duration and intensity of studies, emotionally challenging work and overnight on-call duties with long working hours have been identified as causative factors for sleep deprivation.^5,6,19,20^

For this survey, the LSEQ was chosen instead of the PSQI. Indeed, the PSQI evaluates sleep over the last month and more and therefore gives a general longitudinal assessment, which would not be useful to evaluate sleep disorders during the night prior to HFS. Furthermore, the 2 most impaired domains are the ease of awakening from sleep and the integrity of behaviour following wakefulness, which are not explored by the PSQI. Performance and memorization may be directly impaired by these 2 domains given that 50% of the HFS sessions were scheduled early in the morning.

The average sleep time found in the present study is similar to those found in the literature.^21^ The National Sleep Foundation recommends 7-9 hours for young adults and adults.^22^ Too many hours of sleep or less than those recommended compromise well-being and health. A meta-analysis found that cognitive performance, particularly vigilance and memory, was affected by sleep deprivation.^23^ Some studies suggested sleep deprivation increases anxiety, irritability, and depression scores.^24^ The learners with sleep deprivation had 6 hours of sleep on average and therefore were at risk of suffering from the consequences of the lack of sleep described in these studies.

This is consistent with the residents’ thoughts about the negative impact on HFS performance of sleep disturbance. Previous simulation studies with HFS for critical situations demonstrated that during prolonged wakefulness, residents showed a marked deterioration in the clinical management and a decrease in the non-technical skills associated with impaired teamwork, increased sleepiness, and decreased confidence.^8,18,25^ In the clinical context, these negative effects can globally affect the patient’s safety. Indeed, residents may commit 36% more serious medical errors when they are sleep deprived than in a normal sleep situation.^9^

This multicentre study provides an overall prevalence of sleep disturbance due to HFS. These findings should be taken very seriously as simulation educational tools are becoming more important for medical competence-based education purposes. This study should be used to design appropriate assessment tools. High-fidelity simulation sessions did not include any evaluation; these results need to be adapted and interpreted according to individual context. A future objective for HFS is to help in certification simulation.

In order to optimize both the sleep prior to HFS and the improvement of learning, several ideas are suggested by these results. Firstly, to identify, limit, and prevent inappropriate stress or anxiety factors related to the HFS itself. Secondly, in order to avoid professional stressors, it may be wise to schedule HFS only after days off or during the afternoon. Of note, in the present study, HFS was planned during weekdays and not during the weekend. Therefore, impacts of sleep deprivation on the night before HFS when scheduled at weekend shall be further explored. Moreover, preparing the residents more thoroughly prior to HFS by providing them with more relevant information could reduce anticipatory anxiety and might be an option that should be further explored. Cognitive behavioural therapy for insomnia could be offered to residents with significant sleep disturbance, as its positive effects have been shown to prevent sleep disorders.^26,27^

This study has several limitations. The large cohort was mainly composed of residents in anaesthesiology and intensive care. Therefore, this specific group of residents might have influenced the overall results. One might acknowledge that other specialities of residency could have different sleep deprivation prior HFS and that should be specifically explored. Sleep was not measured using objective sensors. Although difficult to process, collecting repeated continuous measurements during sleep for each resident the night prior to HFS would have been a valuable objective measure to compare with a normal night of sleep. However, a poor correlation has been reported between objective measurements of sleep and the perceived efficiency of sleep by the patients.^28^ A comparative review of the different methods for assessing sleep suggested that subjective self-reports were sensitive to changes in sleep.^29^ Specific questionnaires exploring anticipatory anxiety were not used to assess anxiety as an emotional state related to the situation.

No sleep disturbance comparison was performed between several specific contexts such as HFS, board examination, and first nightshift. Residents were not asked to fill out a sleep diary, and no data on personal events that might affect sleep quality were collected. However, one might assume that a lower prevalence of sleep disturbance for resident controls (without nightshift and HFS) has at least partially addressed this issue. However, the specific causal factor of sleep disturbance has not been clearly explored here. The impact of sleep disorders on the technical and non-technical performances of the residents during the HFS session is not evaluated in this study, but this could be the subject of an upcoming study.

Therefore, understanding the reality of sleep disturbance before HFS and its impact on participants is of major importance. Further studies are now warranted to explore the causal factors of sleep disturbance prior to HFS, in order to limit or prevent performance perturbations.

## Conclusions

Among residents participating in HFS, a high prevalence of change in sleep quality during the night before HFS was noted. As sleep deprivation might lead to a deterioration of performance and learning, strategies to help residents to sleep better prior to HFS shall be further explored. Sleep disturbance prior to HFS might be considered and minimized before organizing summative HFS for professional certification.

## Figures and Tables

**Table 1.** Characteristics of the Residents

**Characteristics **	n = 1112
Female, n	580 (52.4%)
Age, years	26 [25-28]
Height, cm	173 [165-180]
Weight, kg	65 [58-73]
Situation	
In a relationship	539 (49.1%)
Alone	454 (41.4%)
Married	102 (9.3%)
Others	2 (0.2%)
Rotations validated, number*	3 [1-5]
Prior HFS, number	1 [0-3]
Time/last night shift (days)	
0	102 (9.5%)
1-3	269 (25.1 %)
4-5	180 (16.8%)
6-10	257 (24.0%)
>10	263 (24.6%)
Sleep data	
Delay since the last night on duty, days	5 [3-10]
Normal sleep duration, hours	7.1 [0.8]
Normal wakeful period, number	1 [0-1]
Speciality	
Anaesthesiology and intensive care	661 (59.6%)
Pediatric	143 (12.9%)
Surgery	45 (4.1%)
Cardiology	5 (0.5%)
General practitioner	192 (17.3%)
Nephrology	9 (0.8%)
Internists	3 (0.3%)
Pneumology	1 (0.1%)
Gynecology	26 (2.3%)
Others	24 (2.2%)

Values are expressed as n (%), mean (SD), or median (25th-75th) as appropriate.

HFS, high-fidelity simulation; SD, standard deviation.

*The number of rotations for residency in France is from 0 to 10 semesters.

**Table 2.** Univariate Analysis of the Leeds Sleep Evaluation Questionnaire. Disturbance of a Mean of 10 mm Minimum for at least 1 Domain Among the 4 Domains Composing the Leeds Questionnaire

**Variable**	>10 mm: Sleep Disturbance (n = 737)	≤10 mm: No Sleep Disturbance (n = 375)	*P*	**Difference (95% CI)**
Characteristics data				
Female, n	365 (49%)	215 (57%)	.015	
Age, years	26 (25-28)	26 (25-28)	.386	
Height, cm	173 (166-180)	171 (165-180)	.086	
Weight, kg	65 (58-74)	65 (58-72)	.444	
Rotation validated, number	3 (1-6)	3 (1-5)	.217	
Prior HFS, number	2 (0-3)	1 (0-3)	.129	
VAS-anticipatory Anxiety for HFS, mm	41 (25)	34 (22)	<.0001	−7.3 (−4.1 to −10.4)
VAS-HFS impact of sleep disturbance on the upcoming performance, mm	42 (14)	53 (11)	<.0001	−11.5 (−13 to −10)
Sleep data				
Delay since the last night on duty, days	5 (3-10)	5 (2-11)	.737	
Normal sleep duration, hours	7.1 (0.8)	7.1 (0.9)	.628	−0.03 (−0.14 to 0.08)
Normal night-time awakenings, number	0.9 (1.1)	0.8 (1.1)	.345	0.07 (−0.07 to 0.20)
Night before HFS sleep duration, hours	6.4 (1.8)	7.3 (1.3)	<.0001	−0.9 (−1.1 to −0.7)
Night before HFS night-time awakenings, number	1.3 (1.5)	0.7 (0.9)	<.0001	0.58 (0.41 to 0.75)
Night-time spent awake, minutes	12 (25)	6 (14)	.0001	5.5 (2.8 to 8.3)
Dream of the HFS, n	15 (2%)	8 (2%)	.882	
Substance intake with potential effect on sleep, n	97 (13%)	60 (16%)	.233	

Values are expressed as n (%), mean (standard deviation), or median [25th-75th] as appropriate.

HFS, high-fidelity simulation; VAS, Visual Analogue Scale.

**Table 3.** Univariate Analysis of the Leeds Sleep Evaluation Questionnaire

**Variable**	>25 mm Disturbance (n = 298)	≤25 mm Disturbance (n = 814)	*P*	**Difference** **(95%CI)**
VAS-prior anticipatory anxiety of HFS, mm	42 (28)	38 (23)	.013	−4.2 (−0.9 to−7.6)
VAS-HFS impact of sleep disturbance on the upcoming performance, mm	36 (15)	49 (12)	<.0001	−13.5 (−15.2 to −11.8)
Sleep data				
Normal sleep duration, hours	7.0 (0.9)	7.1 (0.8)	.358	−0.05 (−0.17 to 0.06)
Normal night-time awakenings, number	0.9 (1.1)	0.8 (1.1)	.366	0.07 (−0.08 to 0.21)
Night before HFS sleep duration, hours	5.8 (2.0)	7.0 (1.4)	<.0001	−1.2 (−1.4 to −0.9)
Night before HFS night-time awakenings, number	1.6 (1.8)	0.9 (1.2)	<.0001	0.61 (0.43 to 0.79)
Night-time spent awake, minutes	16 (33)	8 (16)	<.0001	7.9 (4.9 to 10.9)

Disturbance of a mean of 25 mm minimum for at least 1 domain among the 4 domains composing the Leeds Questionnaire (severe sleep disturbance). Values are expressed as mean (SD).

HFS, high-fidelity simulation; VAS, Visual Analogue Scale; SD, standard deviation.

**Table 4.** Factors Independently Associated with Disturbance of a Mean of 10 mm Minimum for at least 1 Domain Among the 4 Domains Composing the Leeds Questionnaire

**Variable: **Multivariate Model in Which the Quantitative Measurements Are Considered as Continuous Variables	**Odds Ratio (95% CI)**	*P*
Female	0.68 (0.48-0.97)	.033
Height, cm	0.99 (0.98-1.01)	.490
Prior HFS, number	1.03 (0.98-1.09)	.189
VAS-anticipatory anxiety, mm	1.01 (1.00-1.01)	.004
VAS-HFS performance regarding the quality of sleep, mm	0.92 (0.91-0.94)	<.0001
Female	0.84 (0.59-1.21)	.356
Height, >168 cm	1.26 (0.87-1.81)	.220
Number of prior HFS, >2	1.26 (0.93-1.71)	.132
VAS-anticipatory anxiety, >46 mm	1.72 (1.29-2.29)	.0002
VAS-HFS performance regarding the quality of sleep, >45 mm	0.16 (0.12-0.23)	<.0001

HFS, high-fidelity simulation; VAS, Visual Analogue Scale.
